# Effects of long-term tobramycin inhalation solution (TIS) once daily on exacerbation rate in patients with non-cystic fibrosis bronchiectasis

**DOI:** 10.1186/s12931-022-02243-y

**Published:** 2022-12-03

**Authors:** Lotte C. Terpstra, Josje Altenburg, Inez Bronsveld, Martijn D. de Kruif, Yvonne Berk, Dominic Snijders, Wouter Rozemeijer, Harry G. M. Heijerman, Wim G. Boersma

**Affiliations:** 1 Department of Pulmonary Diseases, Northwest Clinics, Wilhelminalaan 12, 1815 JD Alkmaar, The Netherlands; 2 Department of Pulmonary Diseases, Amsterdam Medical Center, Amsterdam, The Netherlands; 3 Department of Pulmonary Diseases, Utrecht University, Utrecht, The Netherlands; 4 Department of Pulmonary Diseases, Zuyderland Medical Center, Heerlen, The Netherlands; 5 Department of Pulmonary Diseases, Canisius Wilhelmina Hospital, Nijmegen, The Netherlands; 6 Department of Pulmonary Diseases, Spaarne Gasthuis, Hoofddorp, The Netherlands; 7 Department of Medical Microbiology, Northwest Clinics, Alkmaar, The Netherlands

**Keywords:** Bronchiectasis, Tobramycin inhalation solution, Exacerbations

## Abstract

**Background:**

Use of long-term tobramycin inhalation solution (TIS) has been shown beneficial in cystic fibrosis (CF) and earlier findings also suggest a benefit in non-CF bronchiectasis. We investigated the efficacy and safety of maintenance TIS once daily (OD) in frequent exacerbating bronchiectasis patients chronically infected by different pathogens sensitive for tobramycin.

**Objective:**

The primary outcome was the frequency of exacerbations during the 12-month study period. Secondary outcomes were time to first exacerbation, change in lung function and quality of life (QoL), bacterial analysis and safety.

**Materials/patients:**

**In this multicenter RCT patients aged ≥ 18-year-old were included with confirmed bronchiectasis and ≥ 2 exacerbations in the preceding year. Patients were assigned (1:1) to receive TIS or placebo OD for 1-year.:**

**Results:**

58 patients were included of which 52 were analyzed in the mITT analysis. TIS reduced exacerbation frequency with a RR of 0.74 (95% CI 0.49–1.14) (*p* = 0.15). Within the TIS population a decrease in number of exacerbations was found (2; *p* = 0.00), which was also seen in the placebo-treated patients (1.5; *p* = 0.00). In the TIS-treated patients the QoL improved (LRTI-VAS *p* = 0.02 Leicester Cough *p* = 0.02) without additional safety concerns. No differences were found for the other secondary outcomes.

**Conclusion:**

Long-term TIS OD is a safe treatment modality and showed a non-significant reduced exacerbation frequency of 0.74 as compared to placebo in bronchiectasis patients chronically infected by tobramycin sensitive pathogens. TIS OD may be a potential therapeutic strategy in selected patients with bronchiectasis suffering from a high burden of disease.

*Trail registration number:* The BATTLE study was registered at Clinical trials.gov number: NCT02657473. Date: 13 august 2016.

**Supplementary Information:**

The online version contains supplementary material available at 10.1186/s12931-022-02243-y.

## Introduction

Non-cystic fibrosis bronchiectasis is a heterogenous respiratory disease characterized by chronic symptoms of cough and sputum production, with recurrent infections, exacerbations and hospitalizations, resulting in a reduced quality of life (QoL) [1, 2]. Exacerbations are associated with an increased morbidity and mortality, and are more common in patients with chronic bacterial infection. [1, 3–5] Particularly in patients chronically infected with *Pseudomonas aeruginosa,* and to a lesser extent with *Haemophilus influenzae* [1, 6]*.* Reducing the number of exacerbations is the corner stone of long-term disease management, with favourable results for long-term macrolide treatment [7–11]. However macrolide therapy is not always as effective, antibiotic resistance may develop and relapse may occur after discontinuation. In addition, long-term treatment with macrolides is associated with gastro-intestinal side effects, and the risk of QT prolongation, which frequently interacts with other medication [3, 12, 13].

An attractive alternative may be the use of inhaled antibiotics which can provide a consistent deposition of high antibiotic concentrations directly to the site of infection with a lower risk of toxicity or systemic adverse events [8, 14]. In cystic fibrosis (CF) long term inhaled antibiotics have been shown to reduce lung function decline and exacerbations and are part of the standard care in CF with *P. aeruginosa* chronic infection [15–17]. The evidence for inhaled antibiotics in non-CF bronchiectasis is limited, however the bronchiectasis guidelines recommend inhaled antibiotics in patients with *P. aeruginosa* chronic infection [18, 19]. A recently published meta-analysis supports this recommendation, however not much is known about the ideal dosage regimen and duration of treatment, and the preference for a certain type of inhaled antibiotics [8].

For aerosolized tobramycin inhalation solution (TIS) a few small studies were conducted in bronchiectasis patients colonized with *P. aeruginosa* and described a decrease in *P. aeruginosa* density in sputum, with an improvement of the respiratory symptoms. In these studies, the duration ranged from 6 weeks to 13 months, TIS was in the majority given twice daily 28 days on–off, and the most common primary outcome was *P. aeruginosa* density in sputum [20–23]. No data have been published about the effect of maintenance use of TIS once daily (OD) on exacerbation frequency, and especially in bronchiectasis patients with chronic infection by *non- P. aeruginosa* Gram-negative bacteria or *Staphylococcus aureus (S. aureus).*

In the present multicentre randomized controlled trial, effects of longterm toBrAmycin inhalaTion soluTion once daiLy on Exacerbation rate, The BATTLE study, we investigated the effect of TIS OD on exacerbation frequency in bronchiectasis patients during one-year maintenance treatment.

## Methods

### Study design

The BATTLE study was a multicenter, double-blind, placebo-controlled randomized controlled trial, conducted in 6 hospitals in the Netherlands between September 2016 and December 2019. Full details of the study design are available in the Additional file 1. Documented approval from the Independent Ethics Committees and Institutional Review Boards was obtained from all participating centers before start of the study and after the interim analysis.

### Study population

Patients with proven bronchiectasis on HRCT, aged ≥ 18-year-old, chronic respiratory symptoms and at least two respiratory exacerbations treated with antibiotics and/or prednisolone in the preceding year were recruited from the outpatient clinic. All participants had one or more positive sputum cultures for gram-negative pathogens or *S. aureus* in the preceding year, as well as one positive sputum culture with the predefined pathogens at baseline. Patients with known CF, active allergic bronchopulmonary aspergillosis, tuberculosis or non-tuberculous mycobacterial infection were excluded. Other exclusion criteria were the use of maintenance antibiotics, except for maintenance treatment with macrolides if treatment was not initiated within 1 month prior to study entry. We also excluded patients treated with prednisolone > 10 mg per day for > 1 month, and/or patients treated with mucolytics. For safety reasons, patients with chronic renal insufficiency (GFR < 30 ml/min), earlier diagnosed tinnitus, hearing impairment, balance disorders or neuromuscular disorders were excluded. An overview of the complete inclusion and exclusion criteria are shown in Additional file 1.

### Procedures

After the run-in period of 4 weeks, stable bronchiectasis patients were randomly assigned (1:1) to receive TIS 300 mg/5 ml OD or placebo (NaCl 0.9%) OD for 52 weeks by using the InnoSpire Deluxe compressor (Philips Respironics) with a SideSteam Plus nebulizer with filter and mouthpiece.

All patients underwent a tolerance test with spirometry before and after the first dose of the study medication to assess the occurrence of local intolerability. Airway hyperresponsiveness was defined as a decrease in FEV_1_% of predicted of 20% following the study medication and/or saturation < 90%, and/or signs of bronchospasm. Study visits were planned at the outpatient ward every 3 months, and clinical, QoL questionnaire’s, bacteriological and laboratory examinations were performed as well as spirometry. The study medication was delivered from a central pharmacy to each study center in batches, stored at 2–8 C. Medication was dispensed to the patient at each visit for a period of 3 months. The empty ampules were collected at each visit in blinded sachets. Patients were asked to use the diary card weekly to examine the respiratory symptoms and if so, to notice an exacerbation. The final visit was conducted 4 weeks (run-out period) after the end of the treatment period. See Additional file 1 for an overview of the study schedule.

### Outcomes

The primary outcome of the study was the number of exacerbations during the 1-year treatment period. A protocol defined pulmonary exacerbation (PDPE) was defined as the presence of three or more of the following symptoms or signs for at least 24 h: 1. increased cough; 2. increased sputum volume and/or purulence; 3. haemoptysis; 4. increased dyspnoea; 5. increased wheezing; 6. fever (> 38.5 °C) or malaise AND the treating physician agreed that antibiotic and/or prednisolone therapy was required. Secondary outcomes were time to next exacerbation, change in lung function (FVC% predicted, FEV1% predicted) and QoL measurements based on lower respiratory tract infections- visual analogue scale (LRTI-VAS), Quality of Life-Bronchiectasis (QOL-B) and the Leicester cough score [24–26]. In addition, change in biomarkers, liver function and renal function, and bacterial diversity in sputum with the development of tobramycin resistance were evaluated in both groups. Additional safety assessments were assessed in both groups. For an overview of the study assessments see Additional file 1.

### Statistical analysis

Details of the randomization process, sample size calculations and statistical analyses were described at the online Additional file 1. Using an expected baseline exacerbation frequency and effect size, a power analyses was done based on a Poisson regression model. After inclusion and follow up of 50% of the randomized patients an interim analysis was conducted to test the assumptions used in the sample size calculation. The interim analysis revealed that the assumptions made for the sample size calculation were appropriate. However, the drop-out rate was 34%, which was higher than the expected drop-out rate of 30% and made it necessary to increase the sample size by 6 patients.

The primary efficacy analysis was performed on the modified intention to treat (mITT) population. Patients who were randomized but dropped out directly after the tolerance test due to a moderate-severe bronchial obstruction, or during the first 2 weeks of the study treatment, were excluded from the mITT analysis. All randomized patients who received and completed treatment according to the study protocol for at least 9 months were included in the per protocol (PP) analysis. Early termination of the study or incomplete data collection was defined as non-evaluable. Statistical analysis was performed using SPSS version 25. The analyses were performed on the mITT- and the PP-population; discrete variables were presented as counts (percentage) and continuous variables as means with standard deviation (SD) if normally distributed and medians with interquartile range (IQR) if not normally distributed. Between groups differences were tested using the chi- square or Fisher exact test if appropriate in case of nominal or ordinal variables. In case of continuous variables, the student T-test or the Mann–Whitney U Test depending on the distribution was used. The effect of TIS as compared to placebo on exacerbation frequency was analyzed by means of negative binomial regression analysis correcting for the exacerbation rate in the year prior to the study. The association between use of TIS and exacerbation rate was expressed as a rate ratio with 95% confidence interval (RR (95%CI)). Linear mixed model analysis was used to analyze the effects on lung function and QoL over time. Results were presented as differences with p-values. Time to first exacerbation during the treatment period was analyzed using a Cox regression analysis. Resulting associations were presented as hazard ratios with 95% confidence intervals (HR (95%CI). A p-value < 0.05 was considered statistically significant.

## Results

### Patients

**Fig. 1:**
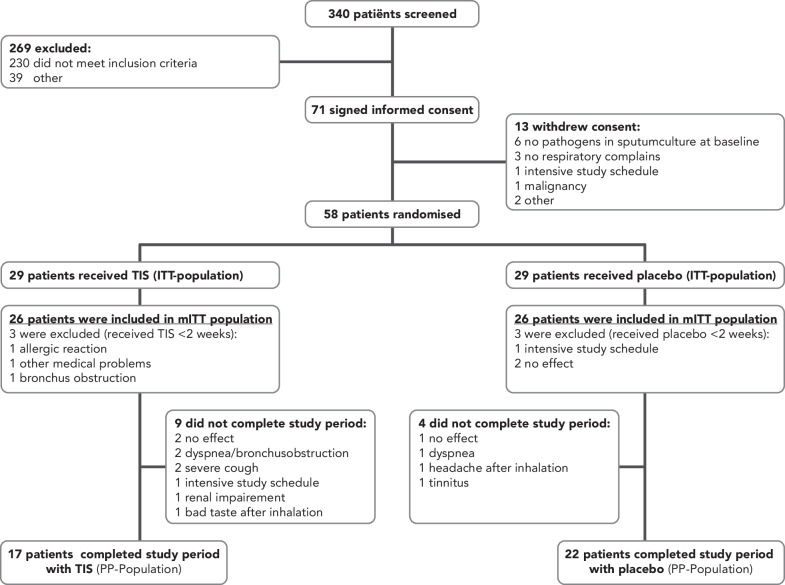
Study flow chart. *TIS* Tobramycin inhalation solution, *ITT* Intention to treat population; *mITT* modified intention to treat population, *PP* per protocol population, Patients were screened in 6 hospitals

A total of 58 patients were randomly assigned to receive either TIS or placebo OD and were included in the ITT-population. A total of 6 patients (3 patients in each group) dropped out in the first 2 weeks of study treatment (Fig. 1). These patients were excluded from the mITT population. Baseline patient characteristics of the mITT population were well balanced between TIS and placebo (Table 1), except for maintenance stable doses of prednisolone < 10 mg, which was significantly more frequent in the TIS population (*p* = 0.04). All patients passed the tolerance test at visit 1, with no occurrence of severe bronchus obstruction (defined as a decrease in FEV_1_% of predicted of > 20% and/or saturation < 90%) after the first dose of the study medication. Treatment compliance was high; from empty ampules count, we estimated that patients adhered 94% over the time in the TIS group and 95% in the placebo group.

**Table 1:** Baseline patient characteristics

	TIS n = 26	Placebo n = 26		TIS n = 26	Placebo n = 26
Age, mean (SD)	67.9 (6.6)	64.1 (14.0)	Inhaled medication, n (%)		
Female, n (%)	13 (50)	17 (65.4)	SABA	15 (57.7)	18 (69.2)
No. exacerbations in the year before study entry, median (IQR)	4 (3–5)	3.5 (2–5.25)	SAMA	3 (11.5)	8 (30.8)
Smoking status, n (%)			LABA	17 (65.4)	18 (69.2)
Current	1 (3.8)	1 (3.8)	LAMA	10 (38.5)	10 (38.5)
Former	13 (50)	15 (57.7)	ICS	17 (65.4)	19 (73.1)
Never	12 (46.2)	10 (38.5)	Mucolytics (hypertonic saline) in the years before study entry	5 (19.2)	4 (15.4)
Etiology, n (%)			Pathogens in sputum at baseline, n (%)		
Post-infective	4 (15.4)	8 (30.8)	*Pseudomonas aeruginosa*	5 (19.2)	9 (34.6)
Idiopathic	6 (23.1)	3 (11.5)	*Haemophilus influenzae*	7 (26.9)	9 (34.6)
COPD	6 (23.1)	6 (23.1)	*Staphylococcus aureus*	4 (15.4)	2 (7.7)
Asthma	3 (11.5)	4 (15.4)	*Streptococcus pneumoniae*	0	1 (3.8)
Immunodeficiency	3 (11.5)	2 (7.7)	Other	10 (38.4)	5 (19.3)
Rheumatic disease	1 (3.8)	1 (3.8)	QoL questionnaires, mean (SD)		
Primary ciliary dyskinesia	0	1 (3.8)	QoL-B Physical	49.8 (30.0)	42.5 (34.8)
Alpha-1 antitrypsin deficiency	0	1 (3.8)	QoL-B Role	64.4 (24.7)	59.2 (23.9)
Yellow nail syndrome	1 (3.8)	0	QoL-B Vitality	53.1 (17.4)	48.7 (17.6)
Aspiration	2 (7.7)	0	QoL-B Emotional	87.9 (10.5)	82.4 (16.5)
Charlson comorbidity index, n (%)			QoL-B Social	65.0 (20.9)	60.1 (23.4)
1 to 2	19 (73.1)	23 (88.5)	QoL-B Treatment Burden	66.4 (17.9)	64.0 (19.4)
3 to 4	7 (26.9)	3 (11.5)	QoL-B Health perceptions	42.4 (16.8)	43.2 (18.9)
Pulmonary function % predicted at baseline, mean (SD)			QoL-B Respiratory symptoms	56.1 (17.8)	57.8 (16.7)
FEV_1_	65.9 (24.9)	70.5 (24.0)	LRTI-VAS Total score	21.0 (7.3)	21.0 (8.5)
FVC	84.4 (20.1)	90.2 (18.6)	Leicester Cough Total score	13.4 (2.9)	13.8 (4.2)
Maintenance AZM, n, (%)	7 (26.9)	4 (15.4)			
Duration of AZM therapy in weeks, median (IQR)	87.8 (3–500)	49.7 (4–100)			
Maintenance prednisolone during the study < 10 mg, n (%)	5 (19.2)	0			
Maintenance immunoglobulin therapy, n (%)	1 (3.8)	1 (3.8)			
Physiotherapy, n (%)	7 (26.9)	8 (30.8)			

Baseline patient characteristics of the modified intention to treat (mITT) population. Data are presented as n (%), mean (SD) or median (IQR). Abbreviations: Tobramycin Inhalation Solution (TIS); Forced expiratory volume in one second (FEV_1_); Forced vital capacity (FVC); Azithromycin (AZM); short acting β agonist (SABA); Short acting anticholinergics (SAMA); long acting β agonist (LABA); long acting anticholinergics (LAMA); inhalation corticosteroids (ICS); Quality of life (QoL); Quality of life bronchiectasis questionnaire (QoL-B); Lower respiratory tract infections—Visual Analogue Scale (LRTI-VAS); Leicester cough questionnaire (Leicester cough)

### Exacerbations

In the mITT population, a total of 99 protocol defined exacerbations were reported during the study, 41 (41.4%) of which occurred in the TIS-treated patients and 58 (58.6%) in the placebo-treated patients. During the study, a median number of 2 (IQR 0–2) exacerbations was found in the TIS treatment group and 2 (IQR 1–3) in the placebo treatment group. Negative binomial regression analysis correcting for baseline exacerbation rate showed a RR of 0.74 (95% CI 0.49–1.14) suggesting a lower, non-significant exacerbation incidence rate in TIS group (*p* = 0.15). For the PP-population similar results in exacerbation incidence were found, with a RR of 0.77 (95% CI 0.42–1.17) (Additional file 1).

The total number of exacerbations of the mITT population also included number of exacerbations during the study period from patients who terminated the study medication.

Additional post-hoc calculations were performed, looking only at exacerbations which occurred during the weeks that patients were on study medication (median 52 (IQR 12–52)). During the use of TIS, the exacerbation rate was reduced by 41% (*p* = 0.02) as compared to placebo.

**Fig. 2:**
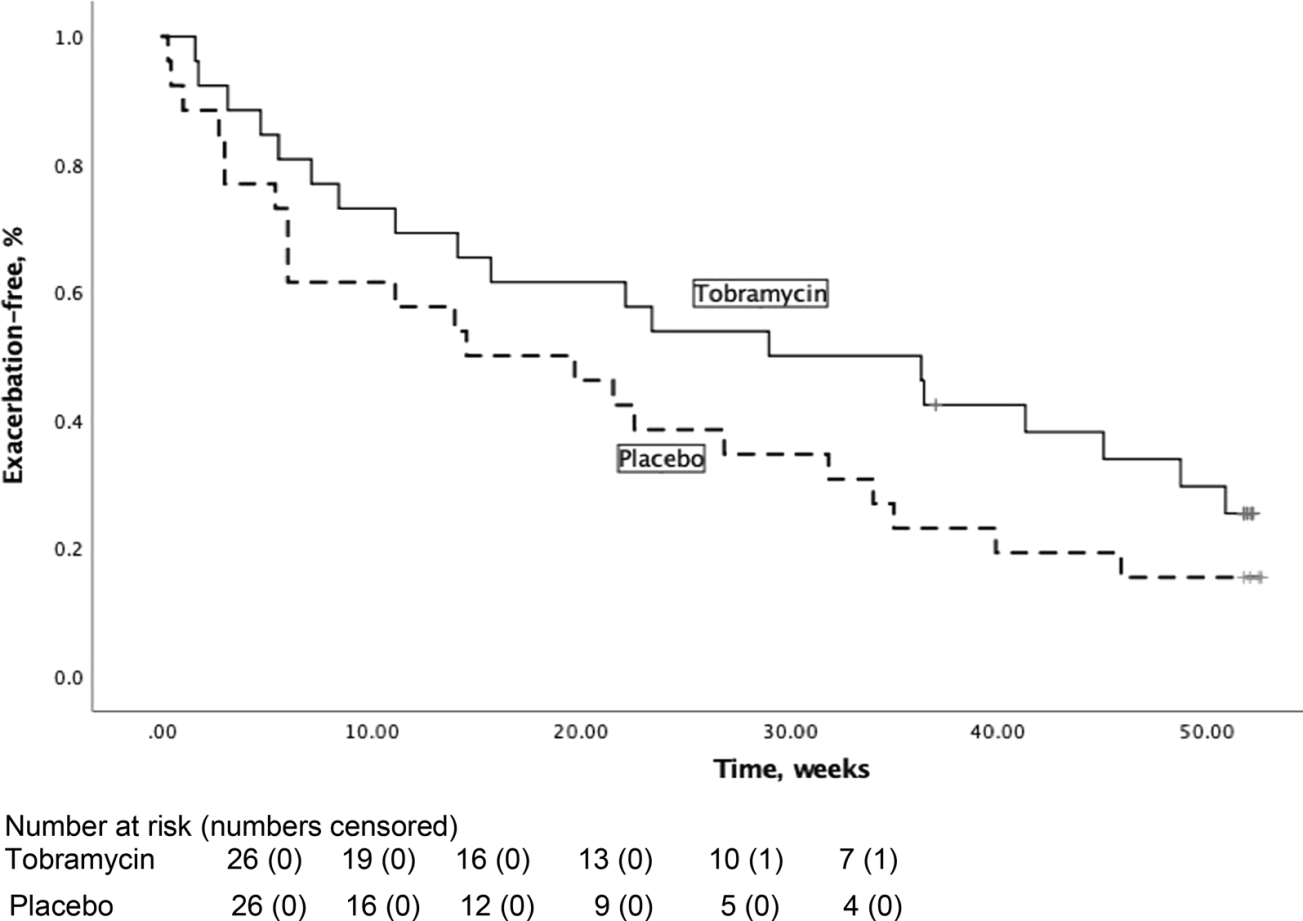
Time to next exacerbation. Kaplan Meier plot of time to fist exacerbation in the mITT population; X-axis: Time in weeks. Y-axis: Percentage of exacerbation free patients. Hazard ratio: 0.64 (95% CI 0.35–1.19)

The secondary endpoint time to first exacerbation differed between both groups with a mean of 29 (SD19.6) weeks for patients treated with TIS and a mean of 21 (SD18.7) weeks for the placebo-treated patients, resulting in a hazard ratio of 0.64 (*p* = 0.15) (Fig. 2).

### Lung function

The longitudinal analysis of lung function in the mITT-population showed no statistically significant differences between both groups (Additional file 1). Comparisons within the TIS treatment group showed a non-significant improvement in FEV_1_% of predicted with a mean of 65.9% (SD 24.9) at baseline and a mean of 72.7% (SD 23) at the end of the study (*p* = 0.58). A non-significant improvement was also found in the placebo population, with a mean of 70.5% (SD 24.0) at baseline and 73.2% (SD 23.5) at the end of the study (*p* = 0.21). The FVC % of predicted was stable during the study for both groups (Table 2).

**Table 2:** Lung function and QoL-questionnaires

Lung function and QoL-questionnaires	TIS			Placebo		
	Start	End	*p-value*	Start	End	*p-value*
Pulmonary function						
FEV_1_% predicted, mean (SD)	65.9 (24.9)	72.7 (23.0)	0.58	70.5 (24.0)	73.2 (23.5)	0.21
FVC% predicted, mean (SD)	84.4 (20.1)	85.0 (24.3)	0.32	90.2 (18.6)	90.3 (19.5)	0.52
QoL-Bronchiectasis questionnaire, mean (SD)						
QoL-B Physical	49.8 (30.0)	47.6 (27.4)	0.75	42.5 (34.8)	42.7 (38.4)	0.47
QoL-B Role	64.4 (24.7)	65.1 (20.7)	0.81	59.2 (23.9)	57.9 (23.9)	0.77
QoL-B Vitality	53.1 (17.4)	51.5 (18.7)	0.85	48.7 (17.6)	45.5 (24.5)	0.25
QoL-B Emotional	87.9 (10.5)	83.3 (11.2)	0.05	82.4 (16.5)	84.1 (12.8)	0.57
QoL-B Social	65.0 (20.9)	64.8 (16.8)	0.81	60.1 (23.4)	67.2 (18.6)	0.10
QoL-B Treatment Burden	66.4 (17.9)	63.9 (24.2)	0.66	64.0 (19.4)	62.8 (21.7)	0.75
QoL-B Health perceptions	42.4 (16.8)	48.1 (19.7)	0.08	43.2 (18.9)	46.1 (17.5)	0.24
QoL-B respiratory symptoms	56.1 (17.8)	61.0 (17.4)	0.05	57.8 (16.7)	59.8 (17.9)	0.14
LRTI-VAS total score, mean (SD)	21.0 (7.3)	18.0 (7.5)	0.02	21.0 (8.5)	20.0 (7.1)	0.18
Leicester Cough total score, mean (SD)	13.4 (2.9)	14.5 (3.2)	0.02	13.8 (4.2)	15.1 (2.6)	0.09

Lung function and QoL questionnaires of the mITT population. Data are presented as mean (SD). A decrease in LRTI-VAS score corresponds to clinical improvement. Tobramycin Inhalation Solution (TIS); Forced expiratory volume in one second (FEV_1_); Forced vital capacity (FVC); Quality of life (QoL); Quality of life bronchiectasis questionnaire (QoL-B); Lower respiratory tract infections—Visual Analogue Scale (LRTI-VAS); Leicester cough questionnaire (Leicester cough)

### Quality of life

The QoL was measured during the study period by using the LRTI-VAS, the Leicester Cough questionnaire, and the QoL-B questionnaire in both groups [24–26]. Longitudinal analysis of the QoL measurements showed no significant improvement for both the mITT and the PP population (Additional file 1).

However, within the TIS population significant improvement of the total LRTI-VAS score (improvement is reflected by a reduction in LRTI-VAS score) and the total Leicester Cough questionnaire score were found after 1-year of study treatment (*p* = 0.02, *p* = 0.02) (Table 2). These differences were not observed into the placebo population (*p* = 0.17; *p* = 0.08) (Table 2). Within groups comparisons showed no significant differences for the 8 QoL-B subscales. However, in the TIS treatment group, the QoL-B respiratory symptom scale (RSS) improved from 56.1 (SD 17.8) to 61.0 (SD 17.4) points after 1-year (*p* = 0.05), with also improvement of the QoL-B health perceptions scale from 42.4 (SD 16.8) to 48.1 (SD 19.7) points at the end of the study (*p* = 0.08), while the QoL treatment burden scale and the emotional scale decreased (*p* = 0.65; *p* = 0.05) (Table 2).

### Microbiology and inflammation

A total of 162 sputum cultures were collected during the study. At baseline, the most common isolated pathogens were *H. influenzae* (16 (30.8%)) and *P. aeruginosa* (14 (26.9%)). At the end of the study, no pathogens were isolated from sputum in 10 (38.5%) TIS-treated patients as compared to 4 (15.4%) placebo-treated patients (Table 3). In 80% of patients with *P. aeruginosa* chronic infection treated with maintenance TIS (n = 5 (19.2%)), no *P. aeruginosa* was isolated in sputum at the end of the study, as compared to 33.3% after placebo (n = 9 (34.6%)). In one (3.8%) TIS-treated patient with *Escherichia Coli (E. coli)* infection tobramycin resistance occurred during the study treatment. After the run-out phase, 4 weeks after the last dose of the study medication, *Aspergillus fumigatus* was isolated from two sputum cultures, 1 (3.8%) in the placebo population and 1 (3.8%) in the TIS population, both with no clinical signs of *Aspergillus* infection. No other ‘opportunistic’ pathogens were found during the study. In addition, serum inflammatory markers were collected every 3 months during the study. No differences were found in CRP, leucocytes, and eosinophil counts. (Additional file 1). During an exacerbation the inflammatory markers were not collected on regulatory base (not shown).

**Table 3:** Microbiological evaluation at the end of the study treatment

Microbiological evaluation	TIS (n = 26)*	Placebo (n = 26)*
No pathogens	10 (38.5)	4 (15.4)
No sputum culture	5 (19.2)	7 (26.9)
*Pseudomonas aeruginosa*	1 (3.8)	6 (23.1)
*Haemophilus influenzae*	3 (11.5)	4 (15.4)
*Staphylococcus aureus*	2 (7.7)	2 (7.7)
*Streptococcus pneumoniae*	1 (3.8)	0
Other gram-negative pathogens	4 (15.4)	3 (11.5)

Microbiological evaluation at the end of the study. Data are presented as n (%). *No significant differences were found between both groups. *TIS* Tobramycin Inhalation Solution

### Safety

All patients underwent a tolerance test at the start of the study. Spirometry measurements were performed before and after the first dose of the study medication (Additional file 1). No occurrence of severe bronchus obstruction was found directly after the first dose. However, 3 (8.8%) TIS-treated patients developed respiratory symptoms in the first 4 weeks of study treatment (none in the placebo-treated patients) and withdrew from the study. Unfortunately, no spirometry was performed during the development of these respiratory symptoms. Two out of 29 (6.8%) TIS-treated patient and 3 out of 29 (10.3%) placebo-treated patients experienced no effect of the study treatment and withdrew from the study. Two out of 58 (3.4%) patients (1 TIS and 1 placebo) mentioned intensive study treatment as a reason for discontinuing the study (Fig. 1). In the mITT population, 7 (26.9%) TIS-treated patients mentioned cough as compared to 5 (19.2%) placebo-treated patients (*p* = 0.47). An overview of all the side effects is shown in Table 4. A total of 28 serious adverse events (SAE) were reported during the study, all related to hospital admissions, of which 24 to a pulmonary exacerbation, 2 patients with known cardiac diseases,1 patient with a near collapse and 1 patient with an anaphylactic reaction on amoxicillin clavulanate. One hundred and fifty-seven adverse events were reported, mostly related to a PDPE or non-PDPE (Additional file 1).

**Table 4:** Side effects

Side effects	TIS (n = 26)*	Placebo (n = 26)*
Cough	7 (26.9)	5 (19.2)
Dyspnea	3 (11.5)	3 (11.5)
Dizziness/headache	2 (7.7)	1 (3.8)
Hoarseness	2 (7.7)	2 (7.7)
Tinnitus	1 (3.8)	1 (3.8)
Dry mouth	2 (7.7)	3 (11.5)
Intensive study schedule	1 (3.8)	1 (3.8)
Swelling lips	1 (3.8)	0
Renal impairment	2 (7.7)	0
Obstipation	1 (3.8)	0
Bad taste after inhalation	1 (3.8)	0

Side effects of the mITT population. Data are presented as n (%). *No significant differences were found between both groups. *TIS* Tobramycin Inhalation Solution

## Discussion

This multicenter, double blinded, randomized controlled trial is the first study with exacerbation frequency as primary outcome in bronchiectasis patients chronically infected by different pathogens and treated with maintenance TIS OD for one year. A non-significant decrease in incidence of exacerbations was found for the TIS-treated patients with a RR of 0.74 (95% CI 0.49–1.14) as compared to placebo. This decrease in number of exacerbations due to TIS in bronchiectasis confirmed the previous published reduced exacerbation frequency of RR 0.81 (0.67–0.97) in a meta-analysis of Laska et al. [8]. However, in this systemic review including 15 randomized controlled trials, different inhaled devices and different types of inhaled antibiotics were analyzed. Resulted in an even more heterogenic bronchiectasis population, which makes comparison with the present study difficult.

In our study, a significant reduction of 2 exacerbations/year was found within the TIS population (*p* = 0.00). However, a similar reduction was noticed in patients receiving placebo treatment, which might be due to the positive effect of saline on sputum evacuation in combination with the known ‘placebo effect’ in randomized controlled trials. This ‘placebo-effect’ may be partially explained by ‘regression to the mean’-effects or may be due to improved bronchiectasis care or adherence during study participation. In addition, inhaled saline as was used by placebo participants is likely to have improved airway clearance. This phenomenon has been described by other authors in the last couple of years and has been hampering other interventional studies in bronchiectasis, especially in RCT’s with inhaled antibiotics [27, 28]. Considering this, our study may have been relatively underpowered since we did not take this substantial placebo-effect into account when designing the study.

In view of the clear trend towards a reduction of exacerbations in the current study, one might assume that our prespecified assumption of a 50% reduction of yearly exacerbations, could have been reached when a larger number of participants had been included. Though, the assumption of a reduction of 50% in number of exacerbations was probably too high in relation to the recent meta-analysis with inhaled antibiotics whereby a rate ratio of 0.81 was observed [8]. On the other hand, a 50% reduction seems clinically relevant because the long-term use of inhaled antibiotics is an intensive and time-consuming therapy and therefore a solid decrease was desirable.

Discontinuation of the TIS treatment was noted in our study population, which influenced our results. When only considering exacerbations while patients where adherent to treatment showed a much larger reduction as compared to placebo-treated patients. However, the reasons for TIS discontinuation were in line with previous studies and presumable reflects real-life adherence in bronchiectasis patients [21, 22].

Differences in effect on lung function were not found, however previous trials showed that lung function is poorly responsive and poorly correlated with other key outcome measures, and they suggested the need to develop biomarkers to identify responders [29].

In our study, the QoL improved, with a significant improvement of the LRTI-VAS total score and the Leicester Cough total score for the TIS population, which was not found for the placebo-treated patients. In addition, the QoL-B measurements showed improvement of the RSS and the health perception scale, with a small decrease of the treatment burden and emotional subscale for the TIS population. This is presumably due to the time-consuming therapy of inhaled antibiotics, which takes about 20 to 30 min a day, and includes the preparation of the InnoSpire, the use of the study medication and the cleaning protocol afterwards.

Other treatment devices, like mesh nebulizers or powder inhalators provide a faster treatment modality and may therefore reduce treatment burden [30]. However, the use of dry powder inhalation is also related to airway irritation, especially in the older and frailer patients, of which the bronchiectasis population mainly consist of [30, 31].

A unique feature of our study is the OD dosing schedule and may have contributed to the high adherence to therapy during study treatment (95% in both groups, based on empty ampules count). Twice daily (BID) on/off dosing every month was originally chosen for TIS with the underlying idea to maximize the treatment effect and to reduce the development of toxic side effects and tobramycin resistance pathogens, however there is a lack of evidence for this specific treatment schedule [32–35]. In addition, previous studies with intravenous administration of aminoglycosides have shown that the OD dosing schedule is equivalent in terms of antimicrobial efficacy as compared to frequent dosing, with an long-term concentration-dependent post-antibiotic effect [36–38]. Our study showed that OD continuous treatment of TIS was well tolerated, with no increase in tobramycin resistance or side effects as compared to earlier trials with the BID 28 days on–off treatment schedule [20–22]. A recently published study in bronchiectasis with *P. aeruginosa* chronic infection showed similar results and described that continuous regimes have advantage over cyclic regimes in reducing *P. aeruginosa* density [31].

In our study safety was monitored closely. All patients underwent a tolerance test at the start of the study, with no observation of severe respiratory symptoms or a decrease in FEV_1_ directly after the tolerance test. However, two TIS-treated patients withdrew from the study in the first two weeks after randomization, one with bronchus obstruction after inhalation and one patient with a local allergic reaction. Patients in the TIS group reported more side effects, which is in line with previous studies with tobramycin [21, 22, 33, 39]. Cough and dyspnea were mentioned mostly during the study, which were reasons for study discontinuation in both groups. No significant differences were found between both groups for the frequently described side effects due to inhaled administration and showed that the use of TIS OD on a continues base can be prescribed safely.

In our study we have focused on all common gram-negative pathogens (and *S. aureus*), causing chronic infection in bronchiectasis, which reflects the daily population of bronchiectasis patients [1, 2]. Tobramycin is a broad-spectrum antibiotic and is expected to affect not only *P. aeruginosa* chronic infection, but chronic infection with other pathogens as well. Indeed, a decrease in all types of pathogens were found for the TIS population at the end of the study, with no significant increase in tobramycin resistance or overgrow of other pathogens. Inhaled tobramycin is currently registered for chronic *P. aeruginosa* infection in CF, but our findings suggest that TIS treatment may also be a treatment option for patients without *P. aeruginosa* chronic infection. However, due to the treatment effect, only a small number of sputum cultures could be obtained at the end of the study, with no significant differences between both groups. Though focusing on the patients with *P. aeruginosa* chronic infection treated with TIS in our population, eradication was achieved in 80% of sputum cultures at the end of the study, as compared to 52% with other pathogens (57% in patients with *Haemophilus influenzae* chronic infection). These results are in line with the previously published studies in CF and non-CF bronchiectasis whereby only patients were included with *P. aeruginosa* chronic infection [31, 39, 40]. However, in our population more patients (9 vs 5 *p* = 0.625) with *P. aeruginosa* chronic infection were randomly included in the placebo population, which may also have led to a limited effect of TIS as compared to placebo.

Another limitation of our study was the small number of included patients based on our power calculation. The earlier mentioned ‘placebo effect’ and the unexpected positive effect of saline nebulization was insufficiently considered in the power analysis, which meant that more patients should have been included. In addition, probably a higher number of patients was required due to the wide range of etiologies in non-CF bronchiectasis [41]. Unfortunately, this makes the study underpowered for evaluating the primary endpoint and resulted in a non-significant reduced exacerbation frequency as compared to placebo.

A strength of our study is the investigator initiated multi center double blinded design, including the heterogenous bronchiectasis population which reflects daily practice, and resulted in an extensive database about this study population.

In conclusion, this is the first study that evaluated the effect of long-term TIS OD on exacerbation frequency in non-CF bronchiectasis patients infected by different pathogens. It showed a non-significant decrease in number of exacerbations with a RR of 0.74 (95% CI 0.49–1.14) as compared to placebo, and an improvement in QoL. Long-term TIS OD was well tolerated with no additional safety concerns. Therefore, TIS OD may be a potential therapeutic strategy in selected patients with bronchiectasis suffering from a high burden of disease.

## Supplementary Information

**Additional file 1.** Study Schedule. Overview of the study schedule. Overview of the in- and exclusion criteria of the BATTLE study. Overview of the in- and exclusion criteria of the BATTLE study. Overview of the study assessments for the BATTLE study. Overview of the primary and secondary endpoints of the study. And in addition, an overview of the safety assessments and additional assessments of the study. Study design BATTLE study. The complete study protocol of the BATTLE study is shown in Additional file 1. Results of the longitudinal analysis, exacerbation, lung function and QoL for the PP-population. Overview of the longitudinal analysis of the primary and secondary endpoints from the per protocol (PP) population. Results of the longitudinal analysis of lung function and QoL for the mITT population. Overview of the longitudinal analysis of the primary and secondary endpoints from the modified intention to treat (mITT) population. Overview of adverse events and serious adverse events. Overview of the adverse events and the serious adverse events during the BATTLE study. Overview of the inflammatory markers in serum. Overview of the inflammatory markers in serum every 3 months during the study visits. During an exacerbation, the inflammatory markers in serum were not measured on regulatory base (not shown).

## Data Availability

The datasets used and/or analyzed during the current study available from the corresponding author on reasonable request.
