# Supplementary material for: Effects of long-term tobramycin inhalation solution (TIS) once daily on exacerbation rate in patients with non-cystic fibrosis bronchiectasis
Source: Respir Res. 2022 Dec 3;23:330. doi: 10.1186/s12931-022-02243-y (PMC9719617; doi:10.1186/s12931-022-02243-y)
Supplement: Supplementary file 1 — Additional file 1. Study Schedule. Overview of the study schedule. Overview of the in- and exclusion criteria of the BATTLE study. Overview of the in- and exclusion criteria of the BATTLE study. Overview of the study assessments for the BATTLE study. Overview of the primary and secondary endpoints of the study. And in addition, an overview of the safety assessments and additional assessments of the study. Study design BATTLE study. The complete study protocol of the BATTLE study is shown in Additional file 1. Results of the longitudinal analysis, exacerbation, lung function and QoL for the PP-population. Overview of the longitudinal analysis of the primary and secondary endpoints from the per protocol (PP) population. Results of the longitudinal analysis of lung function and QoL for the mITT population. Overview of the longitudinal analysis of the primary and secondary endpoints from the modified intention to treat (mITT) population. Overview of adverse events and serious adverse events. Overview of the adverse events and the serious adverse events during the BATTLE study. Overview of the inflammatory markers in serum. Overview of the inflammatory markers in serum every 3 months during the study visits. During an exacerbation, the inflammatory markers in serum were not measured on regulatory base (not shown). [file 12931_2022_2243_MOESM1_ESM.docx]

**Additional file 1**

**
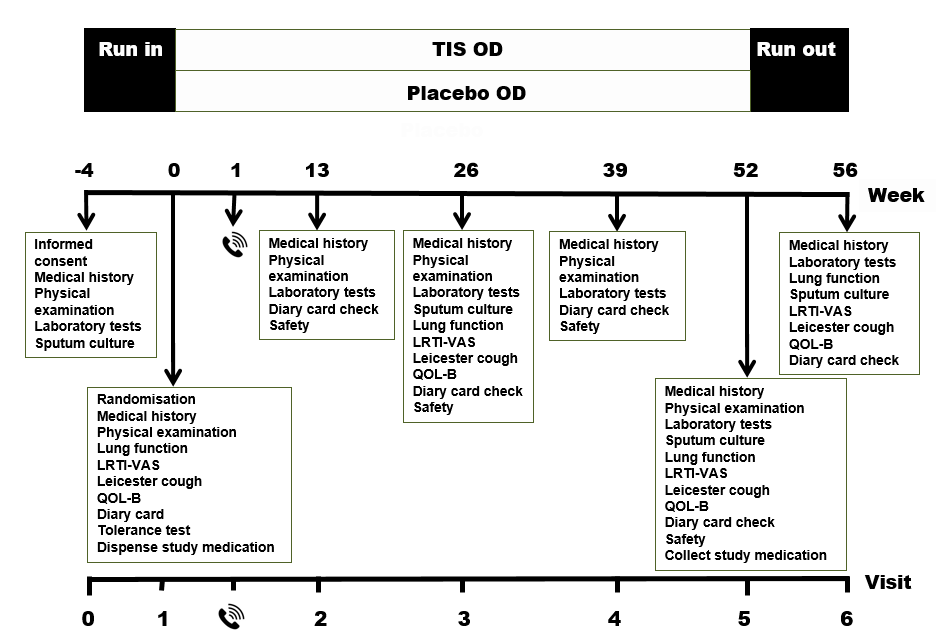
1. Study schedule**

Figure 1. Study schedule. Abbreviations: Tobramycin inhalation solution (TIS); Once daily (OD); Lower respiratory tract infections – Visual Analogue Scale (LRTI-VAS); Leicester cough questionnaire (Leicester cough); Quality of life bronchiectasis questionnaire (QoL-B).

**2. Overview of the in- and exclusion criteria of the BATTLE study**

| **Inclusion criteria** | **Exclusion criteria** |
| --- | --- |
| 1.   Age ≥ 18 years | 1.     Any exacerbation within the month prior to the start of the study |
| 2.   The presence of chronic respiratory symptoms such as cough, dyspnea, expectoration of sputum | 2.     Diagnosis of Cystic Fibrosis |
| 3.   Confirmed bronchiectasis by (HR)CT | 3.     Diagnosis of Active Allergic Bronchopulmonary Aspergillosis |
| 4.   Documented history of at least 2 pulmonary exacerbations treated with courses of antibiotics and/or prednisolone within 12 months before inclusion | 4.     Any oral, IV or inhaled antibiotics (except for macrolides) within 1 month prior to the start of the study |
| 5.   No course of antibiotics or maintenance antibiotics (except for macrolides) 1 month prior to the start of the study | 5.     Any IV or IM corticosteroids or change in oral corticosteroids (> 10 mg) within 1 month prior to the start of the study |
| 6.   Minimal one documented sputum or BAL-fluid culture with gram-negative bacteria or *S. aureus* within 12 months | 6.     Any change/start treatment regimens macrolides, hypertonic saline, inhaled mannitol or other mucolytics, corticosteroids within 1 month prior to the start of the study |
| 7.   Growth of protocol defined pathogens (gram-negative bacteria or *S. aureus*) in sputum at randomization | 7.     Severe immunosuppression or active malignancy |
|  | 8.     Active tuberculosis or non-tuberculous mycobacterial infection |
|  | 9.     Chronic renal insufficiency (eGFR < 30 ml/min) |
|  | 10.  Use of loop diuretics, urea or mannitol |
|  | 11.  Earlier diagnosed hearing impairment, balance disorders or neuromuscular disorders |
|  | 12.  Serious active haemoptysis |
|  | 13.  Have received an investigational drug or device within 1 month prior to the start of the study |
|  | 14.  Serious or active medical or psychiatric illness |
|  | 15.  Pregnancy and childbearing |
|  | 16.  History of poor cooperation or non-compliance |
|  | 17.  Unable to use nebulizers |
|  | 18.  Allergic for tobramycin (or NaCl 0.9%) |

Table 2. In-and exclusion criteria. Abbreviations: Active allergic bronchopulmonary aspergillosis (ABPA); cystic fibrosis (CF); non-tuberculous mycobacterial infection (NTM); High resolution computed tomography (HRCT)

**3. Overview of the study assessments for the BATTLE study**

| **Primary assessment** | **Secondary assessments** |
| --- | --- |
| 1.   Reduce in number of exacerbations | 1.     Time to next exacerbation |
|  | 2.     Change in lung function (FEV1% and FVC%) |
|  | 3.     Change in QoL measurements (QoL-B, LRTI-VAS, Leicester cough) |
| **Safety assessments** | **Additional assessments** |
| 1. Occurrence of any AE or SAE | 1.     Development of tobramycin resistance in sputum (if possible, with MIC values) |
| 1. Occurrence of bronchospasm during the tolerance test | 2.     Bacterial load in sputum and pathogen eradication |
| 1. Occurrence of bronchospasm, dyspnea, cough, or other respiratory symptoms during the study | 3.     Occurrence of new pathogens |
| 1. Occurrence of hearing impairment/ tinnitus | 4.     Change in inflammatory markers in serum |
| 1. Change in safety laboratory values (renal and liver function) | 5.     Analyses of the use of inhaled medication (time consuming, treatment burden) |

Table 3. Overview of the study assessments of the BATTLE study. Abbreviations: Adverse event (AE); Serious adverse event (SAE); non-tuberculous mycobacterial infection (NTM); Forced expiratory volume in one second (FEV1); Forced vital capacity (FVC); Quality of life (QoL); quality of life bronchiectasis questionnaire (QoL-B); Lower respiratory tract infections – Visual Analogue Scale (LRTI-VAS); Leicester cough questionnaire (Leicester cough); Minimum Inhibitory Concentration (MIC);

**4. Study design BATTLE study**

**Objectives**

The primary objective of the study is to determine whether maintenance use of TIS once daily (OD) as compared to placebo may reduce the number of exacerbations per year in patients with bronchiectasis. Secondary objectives are time to next exacerbation, lung function, QoL, laboratory test and microbiological evaluation in patients with bronchiectasis treated with TIS or placebo (supplemental 3).

**Study design**

This study is a prospective, randomized, double-blinded, multicenter, placebo-controlled trial conducted in the Netherlands. The efficacy and safety of TIS OD will be evaluated as compared to placebo during a 52-week treatment period followed by a 4 weeks off-treatment follow up after the last study dose (supplemental 1). Patients with bronchiectasis with recurrent exacerbations (≥ 2 per year) colonized by Gram-negative bacteria or *S. aureus* will be included and evaluated. Subjects meeting all study eligibility criteria (supplemental 2), including a sputum culture with the predefined bacterial pathogens at baseline, are randomized 1:1 to receive OD treatment with either TIS or placebo at visit 1 (week 0). At visit 1 (week 0) a tolerance test will be performed with lung function examination before and after the first dose of the study medication to assess the occurrence of local intolerability. Patients are instructed to use the diary card weekly to examine the respiratory symptoms and if so, to notice an exacerbation. Study visits are planned at the outpatient ward and consists of an up to 4 weeks screening phase (run-in period), a treatment phase of 52 weeks, and a washout phase (run out period) of 4 weeks (supplemental 1). Throughout the study, visits are planned every 3 months, and clinical, QoL questionnaire’s, bacteriological and laboratory examinations will be performed including lung function tests. Documented approval from the Independent Ethics Committees and Institutional Review Boards was obtained from all participating centers before start of the study, according to Good Clinical Practice and local laws and regulations. Written informed consent was obtained from all participants

**Intervention**

TIS 300mg (TEVA pharmaceutics) OD and matched placebo (saline 0.9%) are provided in small plastic ampules of 5ml and are packed in identical sealed boxes and will be dispended on the regular visits every 3 months. The investigators are blinded for the content of the boxes. An OD dosing schedule, and not a twice daily (BID) dosing schedule is administered, which promotes adherence of the relatively intensive and time-consuming treatment schedule, whereby probably no increase in side effects or the development of tobramycin resistance. The study medication is delivered using the InnoSpire Deluxe air compressor (Philips Respironics) with a SideSteam Plus nebulizer with filter and mouthpiece. Salbutamol aerosol with aerochamber is administered every day at a dose of 200mcg before the study medication. The nebulizer will be used for about 10 minutes until the reservoir is empty. The whole procedure: preparation of the nebulizer, inhalation and cleaning takes about 20 minutes to complete. Afterwards the patient should rinse their mouth three times. The study drug administration is performed OD in the morning after completion of the patient’s regular bronchiectasis treatment.

**Study population**

Patients are included with proven bronchiectasis on high resolution computed tomography ((HR)CT), at least two exacerbations in the year prior to the study and a positive sputum culture for gram negative pathogens or *S. aureus* in the preceding year, as well at the screening visit (week 0, visit -4). The inclusion and exclusion criteria for the BATTLE study are shown in Table 1. Patients with known CF, active allergic bronchopulmonary aspergillosis (ABPA), tuberculosis or non-tuberculous mycobacterial infection (NTM) are excluded. All co-medications are allowed, except for any oral (except for macrolides), IV or inhaled antibiotics or corticosteroids (> 10 mg) within 1 month prior to the start of the study. Other exclusion criteria are the use of immunosuppressive agents or any change or start of treatment regimens with macrolides, hypertonic saline, inhaled mannitol or other mucolytics within 1 month prior to the start of the study. Because of the potential interaction with tobramycin the use of loop diuretics or mannitol are prohibited during the study.

**Sample size**

We hypothesize that maintenance treatment with TIS reduces the number of exacerbations per patient by 50%. This reduction seems clinically relevant because long-term use of inhaled antibiotics is an intensive and time-consuming therapy, and therefore a significant decrease in number of exacerbations is desirable. In addition, we assumed that maintenance treatment with TIS OD as well as intermittent TIS BID is comparable to that of maintenance AZM treatment. This assumption is derived from data of the BAT (Bronchiectasis and Long-term Azithromycin Treatment) trial (placebo: mean 2,1 exacerbations (Sd 1.6); azithromycin: mean 0,8 exacerbations (sd 1.1).^23^ The reduction in percentage is used for the determination of sample size. A Poisson regression model is used to determine group size.^24^ For type I error and type II error 0.05 and 0.2 are used respectively. The hypothesis is tested two sided. With a baseline exacerbation rate of 2.1 in the placebo group and an expected response rate ratio of 0.5 with an exposure time of 1 year a total of 18 evaluable patients are required to be on each treatment arm. With a drop-out percentage of 30% we must include totally 48 (24 patients per group) patients.^20,21^ Due to unforeseen reasons 2 extra patients per arm are included. So, a total of 52 patients are included in the study.

**Interim analysis**

After inclusion and follow up of 50% of the randomized patients, an interim analysis will be conducted by an independent statistician blinded for the study. The interim analysis will be used to calculate the predetermined effect size, and whether more patients should be included depending on this effect size. Depending on the frequency of exacerbations at baseline and the effect size, a power analyses will be performed by using the Poisson regression model.^24^

**Randomization**

Block randomization of 4 will be performed centrally with an allocation ratio of 1:1 between groups. The numbers are anonymously dispensed in closed envelopes and stored at the pharmacy of the Northwest Clinics location Alkmaar and at an independent medical doctor. At visit 1 (week 0) patients receive the unique randomization number that allows subsequent identification of their randomized treatment group allocation. The study is blinded for treatment assignment and regimen.

**Safety assessments**

Safety analyses include the occurrence of AE’s and SAE’s, with special interest to bronchospasm, hemoptysis and hypersensitivity reactions. In addition, renal and liver function disorders and the occurrence of hearing impairment and/or tinnitus probably due to the use of TIS will be evaluated. The development of tobramycin resistant pathogens in sputum will be observed in both groups, including the occurrence of NTM and/or *Aspergillus fumigatus.*

A tolerance test will be performed with the first dose of study medication to evaluate the occurrence of inhalation induced bronchospasms. Defined as a decrease in FEV_1_ % of predicted of 20% following the study drug, and/or saturation <90%. A safety analysis will be performed by an independent expert every six months during the study and might recommend termination of the study if there are any safety concerns, outstanding benefit and/or futility.

**Exacerbations**

All participants are provided with 24-hour contact details and invited to contact their respiratory physician or general practitioner and/or study staff when they experience worsening of respiratory signs and symptoms, to ensure that these symptoms are evaluated prospectively. In case of an exacerbation, participants are asked to provide a fresh sputum sample and are instructed to ensure that antibiotic and/or prednisolone prescriptions are provided by the own respiratory physician of the centres or the general practitioner. Criteria for a protocol defined pulmonary exacerbation (PDPE) are adjudicated prospectively by the treating respiratory physician. Deteriorations in respiratory symptoms that do not meet criteria for PDPEs will be termed non–protocol-defined pulmonary exacerbations (non-PDPEs). In these circumstances, participants are advised not require antibiotics, only if clinically indicated determined by the treating physician or the general practitioner. During an exacerbation, the study treatment is continued if possible, unless the study medication is not tolerated, or the exacerbation is believed to be related to the study drug.

**Exacerbation definition**

A PDPE is defined as the presence of three or more of the following symptoms or signs for at least 24 hours:

1. increased cough

2. increased sputum volume and or/purulence

3. haemoptysis

4. increased dyspnoea

5. increased wheezing

6. fever (>38.5°C) or malaise

AND the treating physician agreed that antibiotic and/or prednisolone therapy is required.

**Study discontinuation**

All participants who receive any study medication are encouraged to complete all the study assessments. However, participants can terminate the study at any time for any reason without any consequences. The investigator/treating physician can also decide to withdraw a subject from the study for urgent medical reasons. We estimate a drop-out of 30% patients based on previous studies with inhaled antibiotics

**Efficacy analysis**

Efficacy analysis will be performed in the intention to treat (ITT) population, defined as all randomized patients, and the modified intention to treat population (mITT). The mITT population excludes the randomized patients who dropped out directly after the tolerance test, or in the first two weeks of study treatment (non-evaluable). Analysis in the per protocol population (PP), defined as all randomized patients who received and completed treatment according to the study protocol for at least nine months, will serve as supporting evidence.

**Methods of analysis**

Descriptive statistics for patients treated with TIS or placebo will be calculated at baseline in the ITT population. Discrete variables will be presented as counts (percentage) and continuous variables as means with standard deviation (sd) if normally distributed and medians with interquartile range (IQR) if not normally distributed. Between groups differences will be tested using the students T-test or the Mann-Whitney U Test depending on the distribution. The effect of TIS as compared to placebo on exacerbation frequency will be analyzed by using the Poisson regression analysis. Linear mixed model analysis will be used to analyze the effects on lung function and QoL over the time. The minimal important difference (MID) of the QoL-questionnaires in bronchiectasis is previously reported only for the QoL-B respiratory symptom scale, with an increase of 8 points representing clinical relevance. The MID for the total score of the Leicester cough questionnaire is 1.3 points.^25,27^ Time to first exacerbation during the treatment period, as well as during the wash-out period, will be assessed using Cox proportional hazards regression. A p-value < 0.05 is considered statistically significant. The data will be collected in the online Electronic Case Report Form (ECRF) of Castor EDC – Medical Research and the analysis will be conducted by using IBM SPSS 25 for Windows.

**Supplemental 5. Results of the longitudinal analysis, exacerbation, lung function and QoL for the PP-population**

All randomized patients who received and completed treatment according to the study protocol for at least 9 months were included in the per protocol (PP) analysis. A total of 29 patients were included in the per protocol analysis. 17 (65%) patients were treated with TIS and 22 (84%) patients were treated with placebo. Longitudinal analysis showed a RR of 0.70 (0.42-1.17)

|  |  | **Difference (TIS-placebo) 26 weeks** | **P-value** |  | **Difference (TIS-placebo) 52 weeks** | **P-value** |
| --- | --- | --- | --- | --- | --- | --- |
| **QoL_B_physical** |  | -7.73 | 0.27 |  | -4.44 | 0.51 |
| **QoL_B_role** |  | -13.14 | 0.01 |  | -0.95 | 0.85 |
| **QoL_B_vitality** |  | -5.60 | 0.37 |  | 6.48 | 0.29 |
| **QoL_B_emotional** |  | -0.83 | 0.82 |  | -6.04 | 0.10 |
| **QoL_B_social** |  | -9.58 | 0.07 |  | -2.37 | 0.65 |
| **QoL_B_treatmentburden** |  | 10.53 | 0.22 |  | 0.11 | 0.99 |
| **QoL_B_healthperc** |  | -9.17 | 0.07 |  | 2.98 | 0.55 |
| **QoL_B_resp_sympt** |  | -7.46 | 0.16 |  | -0.53 | 0.92 |
| **Lrti_vas_dyspnoea** |  | 0.31 | 0.68 |  | 0.26 | 0.73 |
| **Lrti_vas_tiredness** |  | -0.44 | 0.54 |  | -0.42 | 0.55 |
| **Lrti_vas_colour_phlegm** |  | -0.82 | 0.34 |  | -0.89 | 0.29 |
| **Lrti_vas_cough** |  | 0.09 | 0.90 |  | -0.52 | 0.49 |
| **Lrti_vas_pain** |  | 0.76 | 0.23 |  | 0.64 | 0.30 |
| **Lrti_vas_total_score** |  | -0.25 | 0.91 |  | -0.64 | 0.77 |
| **Leic_score_physical** |  | -0.20 | 0.54 |  | -0.21 | 0.52 |
| **Leic_score_psych** |  | -0.60 | 0.11 |  | 0.36 | 0.32 |
| **Leic_score_social** |  | -0.72 | 0.05 |  | -0.12 | 0.72 |
| **Leic_score_total** |  | -1.5 | 0.14 |  | -0.35 | 0.72 |

|  |  | **Difference (TIS-placebo) at 26 weeks** | **P-value** |  | **Difference (TIS-placebo) at 52 weeks** | **P-value** |
| --- | --- | --- | --- | --- | --- | --- |
| **FEV1%** |  | -2.19 | 0.35 |  | -3.83 | 0.13 |
| **FVC%** |  | -4.54 | 0.16 |  | -3.16 | 0.35 |

Abbreviations: Forced expiratory volume in one second (FEV1); Forced vital capacity (FVC).

**Supplemental 6. Results of the longitudinal analysis of lung function and QoL for the mITT population**

|  | **Difference (TIS-placebo) at 26 weeks** | **P-value** | **Difference (TIS-placebo) at 52 weeks** | **P-value** |
| --- | --- | --- | --- | --- |
| **FEV1%** | -0.30 | 0.89 | -1.61 | 0.488 |
| **FVC%** | -1.90 | 0.51 | -0.70 | 0.82 |

Abbreviations: Forced expiratory volume in one second (FEV1); Forced vital capacity (FVC).

|  | **Difference (TIS-placebo) at 26 weeks** | **P-value** | **Difference (TIS-placebo) at 52 weeks** | **P-value** |
| --- | --- | --- | --- | --- |
| **QoL_B_physical** | -6.76 | 0.70 | -3.65 | 0.53 |
| **QoL_B_role** | -8.70 | 0.06 | 1.60 | 0.72 |
| **QoL_B_vitality** | -5.64 | 0.31 | 4.07 | 0.47 |
| **QoL_B_emotional** | 0.07 | 0.98 | -4.17 | 0.19 |
| **QoL_B_social** | -8.78 | 0.07 | -4.68 | 0.33 |
| **QoL_B_treatmentburden** | 4.15 | 0.17 | 3.52 | 0.63 |
| **QoL_B_healthperc** | -5.89 | 0.20 | 1.89 | 0.68 |
| **QoL_B_resp_sympt** | -5.14 | 0.24 | 0.07 | 0.99 |
| **Lrti_vas_dyspnoea** | 4.16 | 0.50 | 0.19 | 0.76 |
| **Lrti_vas_tiredness** | -0.76 | 0.23 | -0.49 | 0.43 |
| **Lrti_vas_colour_phlegm** | -0.91 | 0.21 | -1.38 | 0.06 |
| **Lrti_vas_cough** | -0.14 | 0.84 | -0.56 | 0.40 |
| **Lrti_vas_pain** | 0.75 | 0.18 | 0.66 | 0.23 |
| **Lrti_vas_total_score** | -0.35 | 0.86 | -1.38 | 0.48 |
| **Leic_score_physical** | -0.20 | 0.48 | -0.16 | 0.55 |
| **Leic_score_psych** | -0.42 | 0.19 | 0.27 | 0.38 |
| **Leic_score_social** | -0.49 | 0.11 | -0.08 | 0.79 |
| **Leic_score_total** | -1.51 | 0.08 | -0.49 | 0.55 |

Abbreviations: Quality of life (QoL); quality of life bronchiectasis questionnaire (QoL-B); Lower respiratory tract infections – Visual Analogue Scale (LRTI-VAS); Leicester cough questionnaire (Leicester cough).

**Supplemental 7. Overview of adverse events and serious adverse events**

| **Serious adverse events** | **28** |
| --- | --- |
| **Hospital admission** | 28 |
| Protocol defined pulmonary exacerbation | 24 |
| Known cardiac diseases | 2 |
| Near- collapse | 1 |
| Anaphylactic reaction on amoxicillin clavulanate | 1 |
| **Adverse events** | **157** |
| Protocol defined pulmonary exacerbation | 99 |
| Non-protocol defined pulmonary exacerbation | 20 |
| Antibiotics for other reasons | 8 |
| Persistent cough/ dyspnea/ hoarseness | 4 |
| Tinnitus | 3 |
| Headache | 3 |
| Strain muscles | 3 |
| Tiredness | 2 |
| Rectal bleeding/Anemia | 2 |
| Nausea/vomiting | 2 |
| Chest pain | 2 |
| Radiotherapy (treatment malignancy) | 2 |
| Dry mouth | 2 |
| Heart failure | 1 |
| Anaphylactic reaction | 1 |
| Renal dysfunction due to prostate hyperplasia | 1 |
| Renal dysfunction due to diuretics | 1 |
| Renal dysfunction | 1 |

**Supplemental 8. Overview of the inflammatory markers in serum**

| **CRP, mean(sd)** | **Tobramycin** | n (%) | **Placebo** | n (%) | *p-value* |
| --- | --- | --- | --- | --- | --- |
| Start study | 10.2 (9.7) | 26 (100) | 7.3 (10.6) | 26 (100) | **0.02** |
| 3 months | 16.3 (36.7) | 22 (84.6) | 8.0 (14.5) | 26 (100) | 0.26 |
| 6 months | 5.4 (7.2) | 20 (76.9) | 9.5 (13.9) | 23 (88.5) | 0.32 |
| 9 months | 8.5 (12.0) | 14 (53.8) | 6.5 (7.7) | 20 (76.9) | 0.83 |
| End study | 7.9 (11.3) | 19 (73.1) | 11.0 (23.8) | 23 (88.5) | 0.89 |
| Run out (after 4 weeks) | 20.1 (43.8) | 14 (53.8) | 6.5 (6.0) | 18 (69.2) | 0.76 |
|  |  |  |  |  |  |
| **Leucocytes, mean(sd)** | **Tobramycin** | n (%) | **placebo** | n (%) | *p-value* |
| Start study | 10.3 (4.4) | 26 (100) | 9.0 (3.6) | 26 (100) | 0.25 |
| 3 months | 10.6 (4.8) | 22 (84.6) | 9.0 (3.4) | 26 (100) | 0.24 |
| 6 months | 10.6 (4.5) | 20 (76.9) | 9.1 (3.3) | 23 (88.5) | 0.25 |
| 9 months | 9.8 (5.6) | 14 (53.8) | 9.1 (2.4) | 20 (76.9) | 0.61 |
| End study | 10.3 (5.0) | 19 (73.1) | 8.8 (2.4) | 23 (88.5) | 0.52 |
| Run out (after 4 weeks) | 10.6 (5.5) | 14 (53.8) | 9.1 (2.3) | 18 (69.2) | 0.51 |
|  |  |  |  |  |  |
| **Eosinophils, mean(sd)** | **Tobramycin** | n (%) | **Placebo** | n (%) | *p-value* |
| Start study | 0.2 (0.1) | 26 (100) | 0.3 (0.4) | 26 (100) | 0.54 |
| 3 months | 0.2 (0.1) | 22 (84.6) | 0.3 (0.2) | 26 (100) | 0.06 |
| 6 months | 0.3 (0.3) | 20 (76.9) | 0.4 (0.4) | 23 (88.5) | 0.13 |
| 9 months | 0.2 (0.2) | 14 (53.8) | 0.4 (0.6) | 20 (76.9) | 0.23 |
| End study | 0.2 (0.1) | 19 (73.1) | 0.4 (0.4) | 23 (88.5) | **0.02** |
| Run out (after 4 weeks) | 0.2 (0.1) | 14 (53.8) | 0.3 (0.1) | 18 (69.2) | 0.12 |

8. Overview of the inflammatory markers in serum every 3 months during the study visits. During an exacerbation, the inflammatory markers in serum were not measured on regulatory base (not shown*).*
